# Editorial for the Special Issue “Emergency Medicine and Emergency Room Medical Issues”

**DOI:** 10.3390/medicina59020380

**Published:** 2023-02-16

**Authors:** Pierpaolo Di Micco

**Affiliations:** AFO Medicina, UOC Medicina Interna, P.O. Santa Maria delle Grazie, 80100 Pozzuoli, Italy; pdimicco@libero.it

Together with the “law of entropy”, two of the main reasons for the risk of burnout among physicians who work in emergency departments around the world are the variety of clinical issues presented by patients and the absence of guidelines for managing most of them.

For this reason, to be an active worker in the emergency room is one of the most complex types of role experienced in the health system. This Special Issue of *Medicina*, entitled “Emergency medicine and emergency room medical issues”, was planned on this basis, and physicians around world were invited to contribute papers regarding any type of clinical experience in this field.

Of course, this being a Special Issue born in the COVID-19 era, several articles focus on various aspects of the outbreak of this disease. The management of emergency rooms during the outbreak changed all around the world, as protective equipment, isolation rooms and contumacial areas were measures adopted in the first wave of the pandemic [1,2], with continuous and sequential changes in triage systems [3]. In fact, surgical procedures routinely decreased during the first waves of the pandemic, because all intensive areas were used to manage severe COVID-19 cases [4]. Additionally, these simple changes induced, together with all other aspects of the pandemic and its life-threatening potential, psychological stress among emergency room workers [5]. On the other hand, great attention was given to the prognostic markers of adverse outcomes of patients affected by COVID-19 and admitted to emergency departments. The identification of several clinical conditions that represent risks of mortality for patients affected by COVID-19 [6,7], including the identification of several laboratory markers [8,9] that may play similar roles, attracted the attention of all workers in emergency departments. Furthermore, rare complications of treatments for COVID-19, such as spontaneous or ventilation-induced pneumothorax, pneumopericardium, or pneumopericardium, acquired great relevance during the outbreak [10,11]. Moreover, emergency departments adapted their activities to identify and manage the severe complications of vaccinations against SARS-CoV-2, such as vaccine-induced thrombocytopenia with thrombosis [12].

Of course, cardiovascular diseases also occupy a relevant place in research conducted by scholars around the world. Useful information regarding the use of a mobile cloud 12-lead ECG transmission system for patients with acute coronary syndrome that aims to optimize the time for the “first medical contact to ballon (FMCTB)” and revascularization is provided in [13]. On the other hand, the prognostic aspects and post hoc analysis were reported with a focus on several issues. First of all, differences in prognosis between mechanical cardiopulmonary resuscitation and manual cardiopulmonary resuscitation were evaluated using an Asiatic national registry during pandemic [1,14]. Furthermore, the prognostic roles of chronic or other risk factors, such as smoking or chronic kidney disease, in the context of other life-threatening conditions, such as pulmonary embolism or cardiac arrest, were reported [15,16,17,18].

Of course, to perform an etiological diagnosis of a rare infectious disease in the emergency department is very difficult and, when possible, it is frequently associated with atypical clinical manifestations. Several intriguing case reports from this field are reported in the present Special Issue, focusing on the origins of bacterial pericarditis [19,20].

Hematological abnormalities are also a matter of discussion in the daily clinical practice of emergency departments for both children [21] and adults with several comorbidities, such as liver diseases or portal hypertension [22].

Poisoning plays a significant role in several cases. Thus, the identification of rare clinical presentations or side effects of drugs or external agents always represents a difficult clinical scenario in the emergency room [23,24].

Furthermore, several life-threatening diseases such as cardiogenic shock or severe pulmonary embolism, of which clinicians have started to gain substantial clinical experience, are now being treated with extracorporeal membrane oxygenation, which represents a new frontier for emergency departments [25].

However, although acute illnesses affecting non-surgical areas are fascinating, trauma also represents a significant area of hard work in emergency departments. The usefulness of magnetic resonance imaging has been underlined several times in the history of medicine, although this radiological support is unfortunately not available in all emergency departments [26,27]. Conversely, the management of foreign body aspiration is still dependent on the speed of triage systems and number of physicians and nurses available in the specific moment [28].
